# Fabrication and Properties of Zn-3Mg-1Ti Alloy as a Potential Biodegradable Implant Material

**DOI:** 10.3390/ma15030940

**Published:** 2022-01-26

**Authors:** Shuo Zhang, Pengkai Yuan, Xin Wang, Tiebao Wang, Lichen Zhao, Chunxiang Cui

**Affiliations:** Key Laboratory for New Type of Functional Materials of Hebei Province, School of Materials Science and Engineering, Hebei University of Technology, Tianjin 300400, China; zskln1996@163.com (S.Z.); ypk1079420647@163.com (P.Y.); ahaxin@hebut.edu.cn (X.W.); wtb@hebut.edu.cn (T.W.)

**Keywords:** Zn-Mg-Ti alloy, biodegradable metals, mechanical properties, cytotoxicity

## Abstract

A Zn-3Mg-1Ti alloy was fabricated by ultrasonic treatment of Zn-Mg alloy melt using a Ti ultrasonic radiation rod. The microstructure, phase structure, mechanical properties, degradation property, and in vitro cytotoxicity were investigated systematically. The obtained Zn-3Mg-1Ti alloy is composed of the Zn, Mg_2_Zn_11_, and TiZn_16_. Owing to the grain refinement and second phase reinforcement, the mechanical properties of Zn-3Mg-1Ti alloy is improved. In addition, the Zn-3Mg-1Ti alloy exhibits minimal cytotoxicity compared to pure Zn and Zn-1Ti alloy. Electrochemical tests show that the Zn-3Mg-1Ti alloy has an appropriate degradation rate in Hank’s solution.

## 1. Introduction

Biodegradable metal implants have gained much attention owing to their good mechanical properties, excellent biocompatibility, and degradability [1]. After completing the purpose of repair and treatment, it can be completely dissolved in the human body, avoiding injury to the patient from a second operation [2,3]. Recently, biodegradable metals such as Mg, Fe, and Zn have been increasingly accepted as implant materials [4,5,6,7,8,9,10,11]. However, Fe-based alloys and Mg-based alloys have not been able to solve the problem of too slow and too rapid degradation. Among the three candidate metals, Zn and Zn-based alloys have drawn increasing concern due to their moderate degradation rate [12,13,14]. Zinc is an essential nutrient element that participates in the metabolic processes of the human body and is also a constituent element of a variety of enzymes [13,14,15]. Zn affects the regulation of taste, vision immune function, sexual function, and plays a crucial role in protein synthesis, deoxyribonucleic acid synthesis. In addition, Zn can also induce Ca-P deposition, stimulate bone formation, growth, and mineralization. [16,17,18,19,20]. As a biodegradable material with good biocompatibility and an acceptable degradation rate, pure zinc has been intensively investigated by researchers. However, its poor mechanical properties severely limit its application [14,15]. As we all know, alloying can usually improve mechanical properties. Up to now, more than ten kinds of Zn alloy systems have been developed for biomedical applications. Considering biocompatibility, most biomedical zinc alloys contain nutrient elements [21]. For example, Mg [22], Sr [22], Ca [22], Cu [23], Mn [24], Ag [25], Fe [26], and Li [27]. Considering the biocompatibility and mechanical properties, Mg is the preferred alloying element. Mg is an indispensable trace element in the normal life activities and metabolic processes of the human body, and participates in various metabolic activities of the human body, including the formation of bone cells, activation of various enzymes, and protein synthesis [28,29]. So far, many Zn-Mg alloys have been reported, such as Zn-0.8Mg [30], Zn-1Mg [22], Zn-1.2Mg [31], Zn-1.6Mg [32], Zn-3Mg [33], Zn-0.8Li-0.4Mg [34], Zn-1Mg-0.5Ca [35], Zn-3Cu-1Mg [36] alloys. The results show that the Zn-Mg alloy can improve mechanical properties and biocompatibility, but has little effect on the corrosion properties. In particular, the Zn-3Mg alloy has excellent biocompatibility [33]. In addition, Ti and some of its alloys are widely used as implant materials in vivo due to their excellent biocompatibility and mechanical properties [37]. Zn-Ti alloy can improve the mechanical properties and biocompatibility, and promote the degradation of the alloy [38,39]. To date, no researchers have systematically investigated Zn-Mg-Ti alloys. In addition, the ultrasonic treatment process can effectively refine the grains and improve the mechanical properties of the alloy [40].

This paper aims to prepare a Zn-Mg-Ti alloy by ultrasonic treatment with a Ti ultrasonic radiation rod, and the microstructure, phase structure, mechanical properties, degradation property, and cytotoxicity were systematically investigated.

## 2. Materials and Methods

### 2.1. Alloy Preparation

Commercially pure zinc (purity, 99.995%) and pure magnesium (purity, 99.995%) ingots were used as raw materials. A pure titanium rod with a diameter of 30 mm and a length of 130 mm was used as the ultrasonic radiation rod. The ultrasonic radiation rod was first preheated in pure zinc melt (660 °C) for 60 s. At the same time, a certain amount of Mg was dissolved in another zinc melt (600 °C) and 99.999% argon was used as a protective gas to prevent oxidation. The Zn-Mg melt was next ultrasonically treated for 60 s at a frequency of about 19,000 Hz. After ultrasonic processing, the melt was poured into a steel mold (φ20 mm) to obtain a Zn-Mg-Ti alloy. For comparison, pure zinc melt (600 °C) was also ultrasonically treated to obtain a Zn-Ti alloy. The actual chemical compositions of the zinc alloys determined by X-ray fluorescence spectrometer (XRF, ZSX Primus 2) are given in Table 1. Hereafter, the obtained Zn-Mg-Ti alloy is designated as Zn-3Mg-1Ti, the obtained Zn-Ti alloy is designated as Zn-1Ti.

### 2.2. Microstructure and Phase Characterization

The microstructure and morphology of the specimens were characterized by using an optical microscope (Zeiss AxioCam ICc5, Jena, Germany) and a scanning electron microscope (SEM, S-4800, Hitachi Ltd., Tokyo, Japan) with an EDAX energy dispersive spectrometer (EDS). Phase structures of alloys were determined by X-ray diffraction (XRD, Bruker D8 Discover, Rheinstetten, Germany, 6°/min) using Cu K_α_ radiation. The high-resolution transmission electron microscopy (HRTEM) images of the specimens were obtained by transmission electron microscopy (TEM, Titan Themis G2, The Thermo Fisher Scientific, Waltham, MA, USA). The high-angle annular dark-field scanning transmission electron microscopy (HAADF-STEM) images of the specimens were obtained by spherical aberration correction scanning transmission electron microscope (ac-STEM, FEI-Tecnil-Talos-F200, The Thermo Fisher Scientific). Overall TEM foil specimens were treated by a FIB-SEM crossbeam workstation (Helios Nanolab 600i, The Thermo Fisher Scientific).

### 2.3. Mechanical Test

The mechanical property tests of the specimens include hardness test and compressive test. The HMV-2T microhardness tester was used to test the Vickers hardness of the specimens. The load applied for the test was 100 g, and the holding time was 15 s. Under the same condition, each specimen was tested at least five times, and the average value was taken as the microhardness (HV) of the specimen. The compression test was carried out by using a WDW-300 universal tester with a crosshead speed of 1 mm/min at room temperature. Cylindrical specimens 4 mm in diameter and 8 mm long prepared by wire electrical discharge machining were used for compression tests. Since the compliance of the testing machine significantly affects the accuracy of the elastic modulus, the elastic moduli of the specimens were not obtained by the stress-strain curves.

### 2.4. Electrochemical Test

The electrochemical test was carried out in Hank’s solution at 37 °C with an electrochemical workstation (CHI660E, Corrtest Instruments, Wuhan, China). A three-electrode cell method was used for electrochemical measurements. The specimen with 1.0 cm^2^ exposed area was used as working electrode, the reference electrode was a saturated calomel electrode (SCE) and the counter electrode was a graphite rod. The pH value of Hank’s solution [15] was adjusted to 7.4 with HCl solution and NaHCO_3_ solution. The open-circuit potential was first measured in Hank’s solution for 2400 s to reach stability. Then, the potentiodynamic polarization curve was determined at a scanning rate of 1.0 mV/s. The corrosion current density (*I*_corr_) was estimated by linear fit and Tafel extrapolation using cathodic and anodic branches of the polarization curve. The corrosion rate of the specimen was calculated as follows [23,24,41]:

(1) $$CR=K\frac{I_{c\mathrm{orr}}M}{n\rho }$$

where *CR* is corrosion rate, *K* is 3.27 × 10^−3^ mm g μA^−1^ cm^−1^ yr^−1^, *I*_corr_ is the corrosion current density in μA/cm^2^, *M* is the atomic mass fraction, *n* is the number of transfer electron, ρ is the density of specimens.

### 2.5. Cytotoxicity Test

Mouse fibroblasts (L-929) were used to evaluate the cytotoxicity of the specimens. The cytotoxicity test was conducted by an indirect contact method. First, the specimen was immersed in a sterile Petri dish containing 75% absolute ethyl alcohol for 1 h with ultraviolet lamp irradiation. After that, the specimen was air-dried under ultraviolet irradiation, and leached in Roswell Park Memorial Institute (RPMI) 1640 culture solution at 37 °C for 24 h, with the leaching ratio of 0.2 g/mL. Then, L929 cells in the logarithmic phase were digested with 0.25% trypsin and blown into a single cell suspension. The cell concentration was adjusted to approximately 2 × 10^4^ mL^−1^ with RPMI 1640 medium containing 10% calf serum, and 100 µL per well was inoculated into a 96-well plate. The plate was incubated at 37 °C for 24 h in a 5% CO_2_ incubator. After the incubation, the supernatant in each well was cleaned up, and 100 µL of extracts with concentrations of 100%, 50%, 25%, 12.5%, and 6.25% were added to the specimen group, 5% or 10% dimethyl sulfoxide (DMSO) solution was added to the positive control group, and fresh culture solution was added to the cell control group. In, addition, the culture plates were cultured in 37 °C and 5% CO_2_ incubator for 1, 3, and 5 days, respectively. After the culture, 10 µL thiazole blue (MTT) solution of 5 mg mL^−1^ was added to each well, and continued to incubate at 37 °C for 4 h. Then, 180 µL DMSO was added to each well after aspirating the supernatant. Finally, the absorbance (OD) was measured at 570 nm with a microplate reader, the formula of cell proliferation rate (RGR) is as follows [42,43]:

(2) $$RGR=\frac{OD_{specimen}}{OD_{control}}\times 100\%$$

## 3. Results

### 3.1. Microstructures and Phase Structures of the Alloys

**Figure 1:**
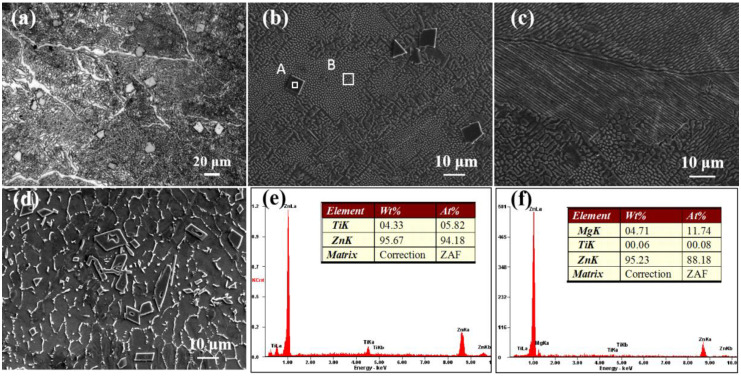
(**a**) Optical micrograph of the Zn-3Mg-1Ti alloy; (**b**,**c**) SEM images of Zn-3Mg-1Ti alloy; (**d**) SEM image of Zn-1Ti alloy; (**e**,**f**) the EDS results of the area A and B in (**b**), respectively.

**Figure 2:**
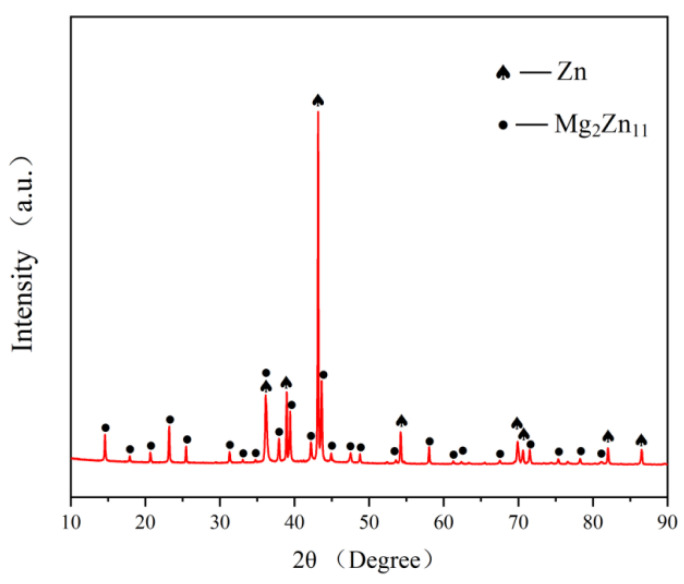
XRD pattern of Zn-3Mg-1Ti alloy.

The metallographic microstructure of the Zn-3Mg-1Ti alloy is shown in Figure 1a. The optical micrograph exhibits that the alloy is mainly composed of laminar and rod-like eutectic mixtures. In addition, some bulks are distributed in the eutectic structures. The SEM images of the Zn-3Mg-1Ti alloy are shown in Figure 1b,c. It can be clearly seen that the morphology of the alloy is basically the same as the result of the optical micrograph. In addition, the eutectic structure has not only a layered structure, but also a rod-like structure. For comparison, the SEM image of the Zn-1Ti alloy is shown in Figure 1d. The grains of α-Zn are separated by white discontinuous network precipitates. Compared with the as-cast pure zinc, the alloy crystal grains are refined [24]. Similar to Zn-3Mg-1Ti alloy, some blocks with regular shapes are embedded in the alloy. Combined with the EDS results (Figure 1e,f), the eutectic structure is rich in Mg and the block-like phase is a Ti-rich phase. Besides, the atomic ratio of Zn and Ti is approximately 16:1. According to the Zn-Mg phase diagram [44], the Zn-Ti phase diagram [45], and the XRD pattern (Figure 2), it can be concluded that the eutectic mixtures are composed of α-Zn and Mg_2_Zn_11_, the Ti-rich phase may be TiZn_16_.

**Figure 3:**
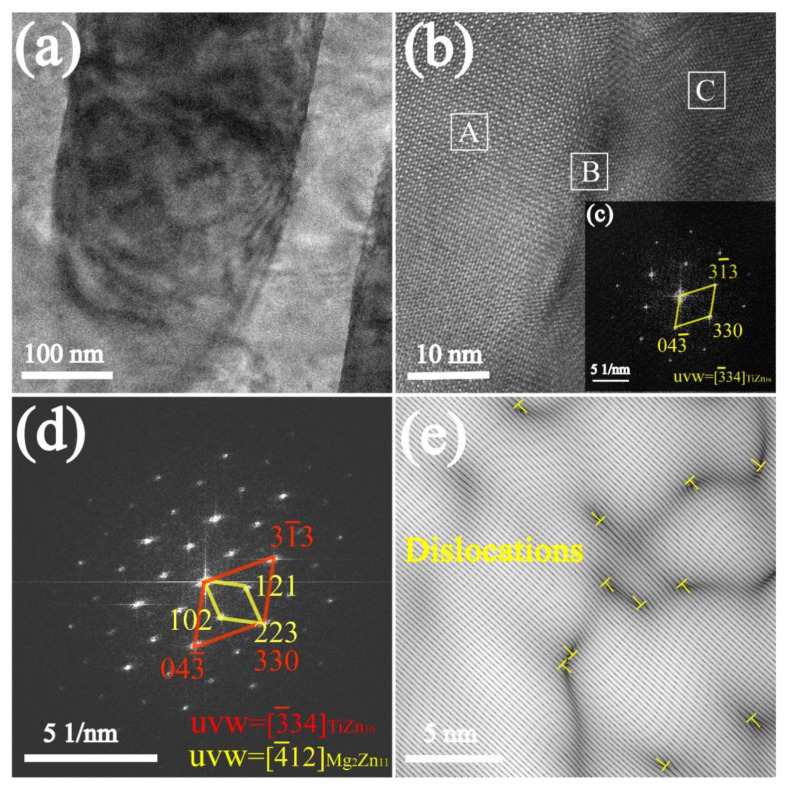
(**a**) TEM image of Zn-3Mg-1Ti alloy; (**b**) HRTEM image of the alloy; (**c**,**d**) FFT electron diffraction patterns of zone C and zone B in (**b**), respectively; (**e**) IFFT image of zone B in (**b**).

To further investigate and qualitative analysis of the Ti-rich phase, the Zn-3Mg-1Ti alloy was observed by TEM. The TEM image of the alloy is shown in Figure 3a. It can be found that the image includes dark areas and bright areas. Figure 3b is the HRTEM image of the specimen, and the image was further filtered by fast Fourier transform (FFT). The FFT electron diffraction patterns of zone C and zone B as shown in Figure 3c,d, respectively. The characterization results show that the HRTEM image contains Mg_2_Zn_11_ phase (zone A), TiZn_16_ phase (zone C), and the TiZn_16_/ Mg_2_Zn_11_ interface (zone B). In addition, the TiZn_16_ phase has an ideal interface matching with the Mg_2_Zn_11_ phase, which is (330)TiZn_16_//(223)Mg_2_Zn_11_. The orientation relationship is a typical coherent interface. It should be noted that some dislocations can also be observed in the interface (Figure 3e).

**Figure 4:**
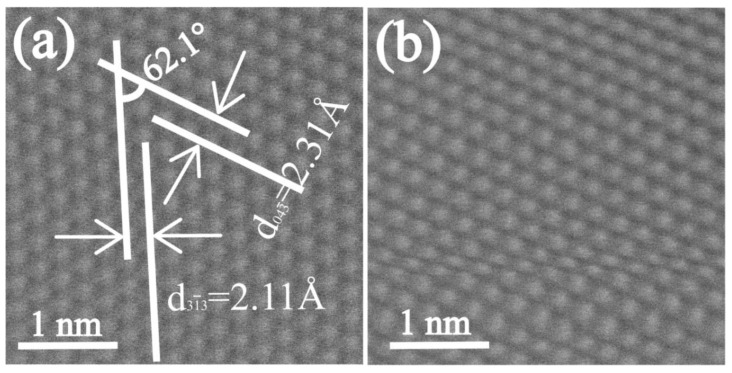
HAADF-STEM images of Zn-3Mg-1Ti alloy: (**a**) no lattice distortion; (**b**) lattice distortion.

Figure 4a,b are the HAADF-STEM images of Zn-3Mg-1Ti alloy. It can be clearly seen that Figure 4a has no lattice distortion. According to the measured results, the interplanar spacing of 2.11 Å and 2.31 Å corresponds to (3$\bar{1}$3) and (04$\bar{3}$) planes of TiZn_16_ (65-2261), respectively. The measured angle between (3$\bar{1}$3) and (04$\bar{3}$) planes is approximately 68.6 °, which is also consistent with the theoretical value. Figure 4b exhibits that the part lattices of TiZn_16_ phase are strongly distorted.

### 3.2. Mechanical Properties of the Alloys

**Figure 5:**
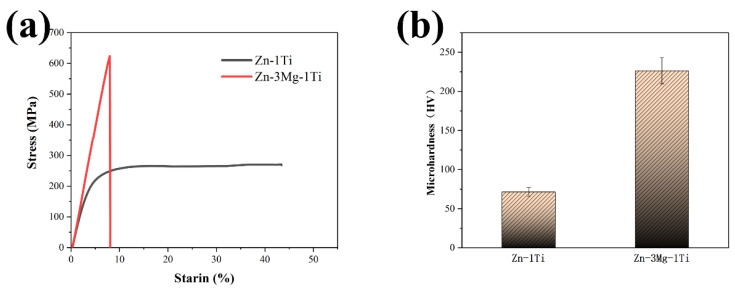
(**a**) Compressive stress–strain curves of specimens; (**b**) microhardness of specimens.

As we all know, the compressive strength (<22 MPa) and microhardness (41 HV) of pure zinc are very low, which cannot meet the requirements of biodegradable implants [15,23]. Figure 5a,b show the compression curves and microhardness histogram of the specimens. It can be clearly seen that after ultrasonic treatment and alloying with Ti, the compressive strength and microhardness of the Zn-1Ti alloy reach 265.1 MPa and 71.3 HV. The Zn-3Mg-1Ti alloy reaches 625.1 MPa and 226.4 HV. The results show that Zn-3Mg-1Ti alloy has more excellent compressive strength and microhardness. However, the plasticity of the Zn-3Mg-1Ti alloy is poor. This may be related to the formation of the intermetallic compound (Mg_2_Zn_11_) in the alloy.

### 3.3. Electrochemical Characterization of the Alloys

**Figure 6:**
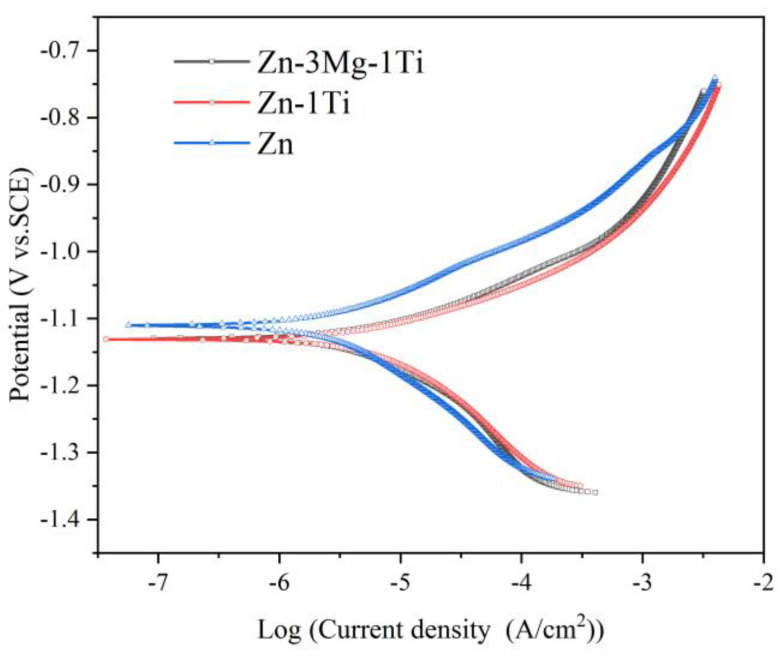
Potentiodynamic polarization curves of the specimens.

Figure 6 shows the potentiodynamic polarization curves of the specimens. The corrosion potentials (*E*_corr_), corrosion current densities (*I*_corr_), and Tafel slopes (*b*_a_ and *b*_c_) directly derived from the polarization curves are listed in Table 2. The calculated polarization resistance (*R*_p_) and corrosion rate are also presented in Table 2. It can be seen that the corrosion potential of Zn-3Mg-1Ti alloy is close to that of Zn-1Ti alloy, but lower than that of pure Zn. However, the corrosion current density of the Zn-3Mg-1Ti alloy is lower than the Zn-1Ti alloy and higher than the pure Zn. After calculation, the corrosion rate of the Zn-3Mg-1Ti alloy (78.5 μm/y) is higher than pure Zn (35.1 μm/y) and lower than the Zn-1Ti alloy (145.9 μm/y).

### 3.4. Cytotoxicity of the Alloys

**Figure 7:**
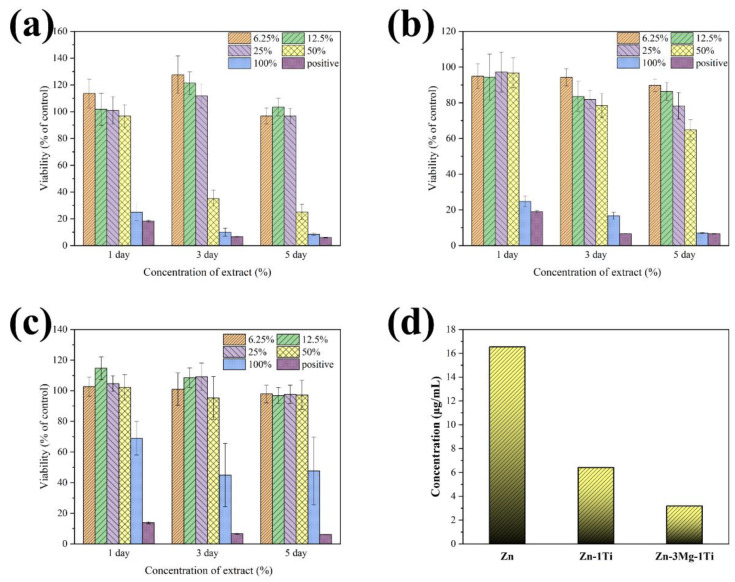
Cell viability after culturing with (**a**) pure Zn, (**b**) Zn-1Ti, (**c**) Zn-3Mg-1Ti extracts for 1, 3, and 5 d; (**d**) Zn^2+^ ion concentration in the extract.

Figure 7a–c shows the RGR of L-929 cells cultured in 6.25%, 12.5%, 25%, 50%, and 100% extracts of pure Zn, Zn-1Ti alloy and Zn-3Mg-1Ti alloy, respectively. The results show that Zn-3Mg-1Ti alloy, Zn-1Ti alloy, and pure Zn cultured in 6.25%, 12.5%, 25% extracts have good cell viability, and the cytotoxicity is basically non-toxic (cytotoxicity grade 0). However, with the increase of cell extract culture concentration and culture time, the RGR in pure Zn decreased dramatically and the cytotoxicity increased to severe toxicity (cytotoxicity grade 3–4). After being cultured in 50% extracts for 5 days, the Zn-1Ti alloy showed a slight decrease in cell activity (cytotoxicity grade 1–2), but when cultured in 100% extracts, the cytotoxicity increased significantly (cytotoxicity grade 4). Nevertheless, after being cultured in 100% extract for 5 days, the cytotoxicity of the Zn-3Mg-1Ti alloy is still slightly toxic (cytotoxicity grade 2). The results suggest that the biocompatibility of Zn-3Mg-1Ti alloy is better than that of Zn-1Ti alloy and pure Zn. In addition, the measured Zn^2+^ ion concentration in the extract of the Zn-3Mg-1Ti alloy is (3.1792 μg/mL), which is the lowest among these specimens (Figure 7d).

## 4. Discussion

### 4.1. Influence of Ultrasonic Treatment

**Figure 8:**
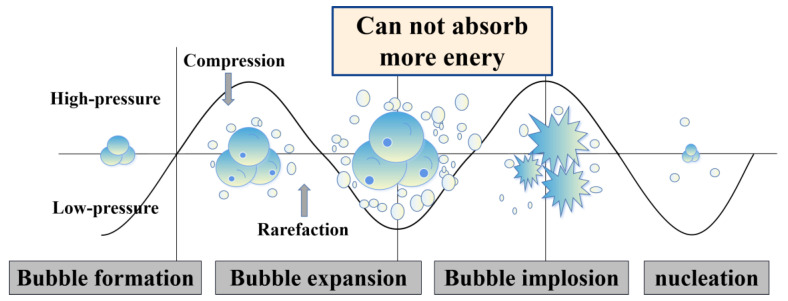
Schematic diagram of ultrasonic cavitation principle.

After ultrasonic treatment, Ti element was introduced into the Zn and Zn-Mg melts by diffusion. This can solve the problem that Zn alloy containing Ti is not easy to be prepared because the melting point of Zn (419.53 °C) is too different from that of Ti (1668 °C). Besides, ultrasonic treatment can not only introduce Ti element, but also reduce the casting defects and specific gravity segregation [40,44]. Not only that, as shown in Figure 8, when the zinc melt is ultrasonically processed, the sound waves propagating into the melt medium will generate alternating high-pressure (compression) and low-pressure (rarefaction) cycles, and the cavitation bubbles will undergo a series of dynamic processes of nucleation, growth, and collapse. The tremendous energy and high-pressure shock wave generated by this cavitation effect will break the primary crystals in the zinc alloy melt and increase the number of heterogeneous crystal nuclei. These nucleation particles spread to all areas of the melt driven by the sound flow, which greatly increases the probability of equiaxial crystal nuclei in the melt [45,46,47].

### 4.2. Microstructures and Mechanical Properties of the Alloys

According to the Zn-Ti phase diagram [45], when the melt temperature reaches 418.6 °C, the eutectic reaction of $L\to \alpha -Zn+TiZn_{16}$ will occur in the melt. Zn and Ti will form the TiZn_16_ phase, and some eutectic structures are distributed on the grain boundaries, which significantly refine the grains [38,48]. Compared with the Zn-1Ti alloy, the morphology of Zn-3Mg-1Ti alloy changed correspondingly with the addition of Mg. Zn-3Mg-1Ti alloy is mainly composed of bulky TiZn_16_ phase and eutectic mixture of α-Zn and Mg_2_Zn_11_. The phase diagram of Zn-Mg shows that eutectic reaction will occur when the temperature is 364 °C and the mass fraction of Mg is 3 wt.% ($L\to \alpha -Zn+Mg_{2}Zn_{11}$). Therefore, the diffraction peaks of Zn and Mg_2_Zn_11_ can be observed in the XRD pattern of the Zn-3Mg-1Ti alloy (Figure 2). However, due to the low content of the Ti element and fast scanning speed, TiZn_16_ was not shown in the XRD pattern. In addition, the TEM test confirmed the existence of TiZn_16_ phase and found that Mg_2_Zn_11_ and TiZn_16_ had an excellent orientation relationship.

The compressive strength and microhardness of the Zn-1Ti alloy (265.1 MPa, 71.3 HV) far exceed pure Zn. This is because the addition of Ti refines the grains of the alloy and produces the second phase of TiZn_16_. According to the Hall-Petch relationship [49], the smaller the grains of the alloy, the higher its strength. In addition, the second phase strengthening also plays a positive role. When the Mg element is introduced to form a ternary alloy, large amounts of eutectic structures are produced. The compression strength and microhardness of Zn-3Mg-1Ti alloy (625.1 MPa, 226.4 HV) are higher than that of Zn-1Ti alloy, which might be attributed to the increase of the eutectic mixtures. Mg_2_Zn_11_ is a hard and brittle phase, which will significantly improve the hardness and strength of the alloy, but inevitably sacrifice a part of plasticity. Furthermore, based on the edge-by-edge matching model [50,51], the mismatch between Mg_2_Zn_11_ phase and TiZn_16_ phase is calculated to be 3.7%, which is less than 6%. In addition, the Mg_2_Zn_11_ phase and TiZn_16_ phase have a coherent orientation relationship, resulting in a strong combination between Mg_2_Zn_11_ and TiZn_16_. The load can be efficiently transferred from one phase to another reinforcing phase so that the hard phase with higher strength can share the load and improve the strength and hardness of the alloy [52]. The lattice distortion is also one of the factors for the improvement of mechanical properties. Therefore, the synergistic effects of grain refinement strengthening, second phase strengthening, lattice distortion and load transfer jointly improve the mechanical properties of the alloy.

### 4.3. Corrosion Behavior of the Alloys

After adding Ti, the corrosion rate of the alloy is significantly increased. This is due to the potential difference between TiZn_16_ phase and α-Zn in the Zn-1Ti alloy, and galvanic corrosion occurred in Hank’s solution, thereby accelerating the corrosion process of the Zn alloy. The potential of the TiZn_16_ phase is lower than that of α-Zn, and it is usually regarded as the anode of micro-battery to be preferentially corroded. Both the Ti-rich phase and the Mg-rich phase will undergo galvanic corrosion with the zinc matrix and be preferentially corroded, thereby accelerating the degradation rate of the alloy. However, Yao et al. [53] and Yang et al. [54] reported that the Mg_2_Zn_11_ phase will reduce the electrode potential difference and inhibit the progress of corrosion. Therefore, the degradation rate of Zn-3Mg-1Ti alloy with Mg_2_Zn_11_ phase is lower than that of Zn-Ti.

### 4.4. Cytotoxicity Assessment of the Alloys

Cytotoxicity evaluation showed that the cell activity of the Zn-3Mg-1Ti alloy was better than that of the Zn-1Ti alloy, while pure zinc has the worst cell activity. The results can be attributable to the Zn^2+^ concentration of the extract. It is well-known that excessive local Zn^2+^ will promote cell death [55]. Our experimental results show that the order of the Zn^2+^ concentration in the leaching solution is as follows: Zn-3Mg-1Ti alloy < Zn-1Ti alloy < pure Zn. Although the degradation rate of Zn-3Mg-1Ti alloy is higher than that of pure zinc and lower than that of Zn-1Ti, it releases the least Zn^2+^ ions. This is due to the TiZn_16_ phase acting as a sacrificial anode to protect the cathode, which reduces the release rate of Zn^2+^ ion, and a large amount of Mg_2_Zn_11_ precipitates in the alloy which will reduce the degradation rate of the alloy [53]. Not only that, both Ti and Mg elements are beneficial to the human body and have important physiological functions in the human body. Combining the above reasons, Zn-3Mg-1Ti alloy has very low cytotoxicity and excellent biocompatibility.

## 5. Conclusions

In this study, the Zn-3Mg-1Ti alloy was fabricated by ultrasonic treatment with a Ti ultrasonic radiation rod. The microstructure, phase structure, compressive strength, microhardness, degradation rate, and cytotoxicity of the alloy were studied. The main conclusions are as follows:

1. Ultrasonic treatment with Ti rod can introduce Ti element, refine the grains of the alloy, and promote the number of heterogeneous nucleation cores;
2. Zn-3Mg-1Ti alloy is mainly composed of Mg_2_Zn_11_, TiZn_16_, and α-Zn. The compressive strength and microhardness of the alloy are excellent. But the alloy is relatively brittle and has poor plasticity;
3. The electrochemical test shows that the Zn-3Mg-1Ti alloy has a suitable degradation rate and is a very promising biodegradable implant material;
4. The Zn-3Mg-1Ti alloy exhibits minimal cytotoxicity and excellent biocompatibility.

In a word, the Zn-3Mg-1Ti alloy has high application potential as a biodegradable implant. We will further improve the plasticity of the alloy and optimize its comprehensive properties in future research.

## Figures and Tables

**Table 1:** Chemical compositions of the specimens.

Specimens	Mg (wt.%)	Ti (wt.%)	Zn (wt.%)
Zn-1Ti	0	1.01	Bal.
Zn-3Mg-1Ti	3.03	0.94	Bal.

**Table 2:** Electrochemical parameters of Zn-3Mg-1Ti, Zn-Ti alloy and pure Zn.

Specimens	*E*_corr_/V_SCE_	*I*_corr_ (μA/cm^2^)	*b*_a_ (mV/dec)	*b*_c_ (mV/dec)	*R*_p_ (kΩ/cm^2^)	Corrosion Rate(μm/y)
Zn	−1.110	2.35	70.0	130.9	8.44	35.1
Zn-1Ti	−1.131	9.77	79.4	178.4	2.44	145.9
Zn-3Mg-1Ti	−1.130	5.26	80.7	125.9	4.06	78.5

## Data Availability

The data presented in this study are available on request from the corresponding author.
